# Supplementary material for: The Expression Profile and Prognostic Significance of Metallothionein Genes in Colorectal Cancer
Source: Int J Mol Sci. 2019 Aug 7;20(16):3849. doi: 10.3390/ijms20163849 (PMC6721156; doi:10.3390/ijms20163849)
Supplement: Supplementary file 1 [file ijms-20-03849-s001.zip › CRC-MTs-2019-08-03-IJMS form-IJMS form-Supplement.pdf]

Title: The Expression Profile and Prognostic Significance of Metallothionein Genes in Colorectal Cancer

Authors: Kuo-Chen Hung[1], Tsui-Chin Huang[2, 3,†], Chia-Hsiung Cheng[4,†], Ya-Wen Cheng[3,5,6,7], Ding-Yen Lin[2,8], Jhen-Jia Fan[9,10], Kuen-Haur Lee[2,3,11,*]

[1]Division of Gastroenterologic Surgery, Department of Surgery, Yuan's General Hospital, Kaohsiung, Taiwan

[2]Ph.D. Program for Cancer Molecular Biology and Drug Discovery, College of Medical Science and Technology, Taipei Medical University, Taipei, Taiwan

[3]Graduate Institute of Cancer Biology and Drug Discovery, College of Medical Science and Technology, Taipei Medical University, Taipei, Taiwan

[4]Department of Biochemistry and Molecular Cell Biology, School of Medicine, College of Medicine, Taipei Medical University, Taipei, Taiwan

[5]Cancer Center, Taipei Medical University Hospital, Taipei Medical University, Taipei, Taiwan

[6]Translational Cancer Research Center, Taipei Medical University, Taipei, Taiwan

[7]Department of R&D, Calgent Biotechnology Co., Ltd, Taipei, Taiwan

[8]Department of Biotechnology and Bioindustry Sciences, College of Bioscience and Biotechnology, National Cheng Kung University, Tainan, Taiwan

[9]Institute of Biochemical Sciences, National Taiwan University, Taipei, Taiwan

[10]Food and Drug Administration, Ministry of Health and Welfare, Taipei, Taiwan

[11]Cancer Center, Wan Fang Hospital, Taipei Medical University

[†]These authors contributed equally to this work.

*Corresponding author: Dr. Kuen-Haur Lee, Graduate Institute of Cancer Biology and Drug Discovery, College of Medical Science and Technology, Taipei Medical University, No. 250 Wu-Hsing Street, Taipei 11031, Taiwan. Tel: +886-2-27361661 ext. 7627, Fax: +886-2-66387537, E-mail: khlee@tmu.edu.tw

Supplemental Information includes one Supplemental Table and 6 Supplemental Figures

Supplementary Information

Figure S1. Survival analysis of CRC patients with SurvExpress (*n*=350). High expression of MT1B (**A**), MT1F (**B**), MT1G (**C**), MT1H (**D**), MT1L (**E**), and MT1X (**F**) were correlated with low risk and good prognosis of CRC patients.

Figure S2. Kaplan–Meier curves according to any two-gene models. Clinical outcomes for the combinations of MT1B/MT1F (**A**), MT1B/MT1G (**B**), MT1B/MT1H (**C**), MT1B/MT1L (**D**), MT1B/MT1X (**E**), MT1F/MT1G (**F**), MT1F/MT1H (**G**), MT1F/MT1L (**H**), MT1F/MT1X (**I**), MT1G/MT1H (**J**), MT1G/MT1L (**K**), MT1G/MT1X (**L**), MT1H/MT1L (**M**), MT1H/MT1X (**N**), and MT1L/MT1X (**O**) mRNA status of CRC patients.

Figure S3. Kaplan–Meier curves according to any three-gene models. Clinical outcomes for the combinations of MT1B/MT1F/MT1G (**A**), MT1B/MT1F/MT1H (**B**), MT1B/MT1F/MT1L (**C**), MT1B/MT1F/MT1L (**D**), MT1B/MT1G/MT1H (**E**), MT1B/MT1G/MT1L (**F**), MT1B/MT1G/MT1X (**G**), MT1B/MT1H/MT1L (**H**), MT1B/MT1H/MT1X (**I**), MT1B/MT1L/MT1X (**J**), MT1F/MT1G/MT1H (**K**), MT1F/MT1G/MT1L (**L**), MT1F/MT1G/MT1X (**M**), MT1F/MT1H/MT1L (**N**), MT1F/MT1H/MT1X (**O**), MT1F/MT1L/MT1X (**P**), MT1G/MT1H/MT1L (**Q**), MT1G/MT1H/MT1X (**R**), MT1G/MT1L/MT1X (**S**), and MT1H/MT1L/MT1X (**T**) mRNA status of CRC patients.

Figure S4. Kaplan–Meier curves according to any four-gene models. Clinical outcomes for the combinations of MT1B/MT1F/MT1G/MT1H (**A**), MT1B/MT1F/MT1G/MT1L (**B**), MT1B/MT1F/MT1G/MT1X (**C**), MT1B/MT1F/MT1H/MT1L (**D**), MT1B/MT1F/MT1H/MT1X (**E**), MT1B/MT1F/MT1L/MT1X (**F**), MT1B/MT1G/MT1H/MT1L (**G**), MT1B/MT1G/MT1H/MT1X (**H**), MT1B/MT1G/MT1L/MT1X (**I**), MT1B/MT1H/MT1L/MT1X (**J**), MT1F/MT1G/MT1H/MT1L (**K**),

MT1F/MT1G/MT1H/MT1X (**L**), MT1F/MT1H/MT1L/MT1X (**M**), and MT1G/MT1H/MT1L/MT1X (**N**) mRNA status of CRC patients..

Figure S5. Kaplan–Meier curves according to any five- and six-gene models. Clinical outcomes for the combinations of MT1B/MT1F/MT1G/MT1H/MT1L (**A**), MT1B/MT1F/MT1G/MT1H/MT1X (**B**), MT1B/MT1F/MT1G/MT1L/MT1X (**C**), MT1B/MT1F/MT1H/MT1L/MT1X (**D**), MT1B/MT1G/MT1H/MT1L/MT1X (**E**), MT1F/MT1G/MT1H/MT1L/MT1X (**F**), and MT1B/MT1F/MT1G/MT1H/MT1L/MT1X (**G**) mRNA status of CRC patients.

Supplementary Table 1. Top 20 down-regulated genes in CRC

T/N ratio	GENE_SYMBOL	GENE_NAME
0.01	GUCA2B	guanylate cyclase activator 2B (uroguanylin)
0.03	B3GNT7	UDP-GlcNAc:betaGal beta-1,3-N-acetylglucosaminyltransferase 7
0.05	CA7	carbonic anhydrase VII
0.14	B3GNT7	UDP-GlcNAc:betaGal beta-1,3-N-acetylglucosaminyltransferase 7
0.14	MUC4	mucin 4, cell surface associated
0.15	MT1F	metallothionein 1F
0.20	MT1G	metallothionein 1G
0.21	LOC344887	NmrA-like family domain containing 1 pseudogene
0.21	MT1X	metallothionein 1X
0.21	MT1H	metallothionein 1H
0.22	MT1B	metallothionein 1B
0.22	MT1L	metallothionein 1L (gene/pseudogene)
0.24	SLC35C2	solute carrier family 35, member C2
0.24	UBE2H	ubiquitin-conjugating enzyme E2H
0.24	ADAMDEC1	ADAM-like, decysin 1
0.24	ITM2A	integral membrane protein 2A
0.24	MIR22HG	MIR22 host gene (non-protein coding)
0.25	LOC100507053	uncharacterized LOC100507053
0.25	KLF4	Kruppel-like factor 4 (gut)
0.26	SEMA6D	sema domain, transmembrane domain (TM), and cytoplasmic domain, (semaphorin) 6D
0.26	PDE4D	phosphodiesterase 4D, cAMP-specific

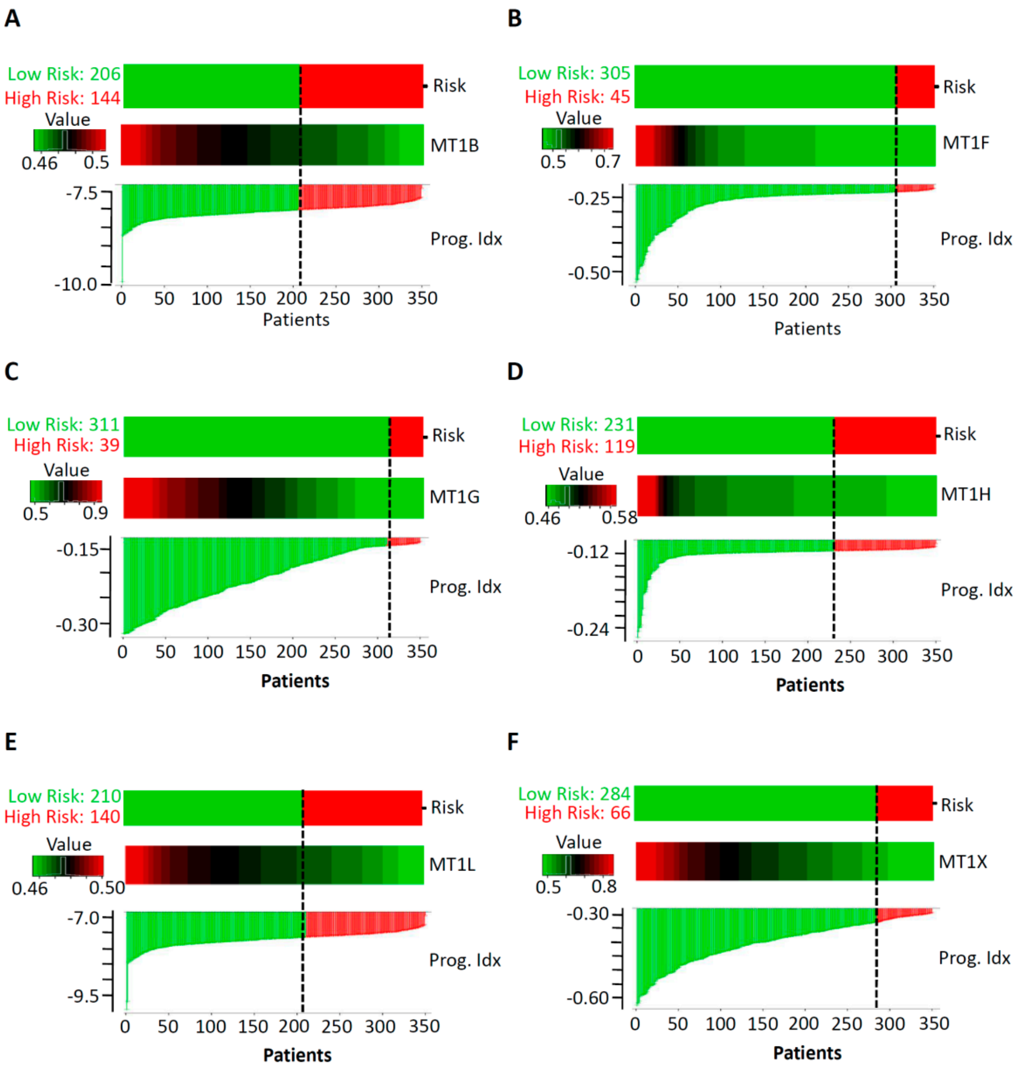

Supplementary Fig. 1

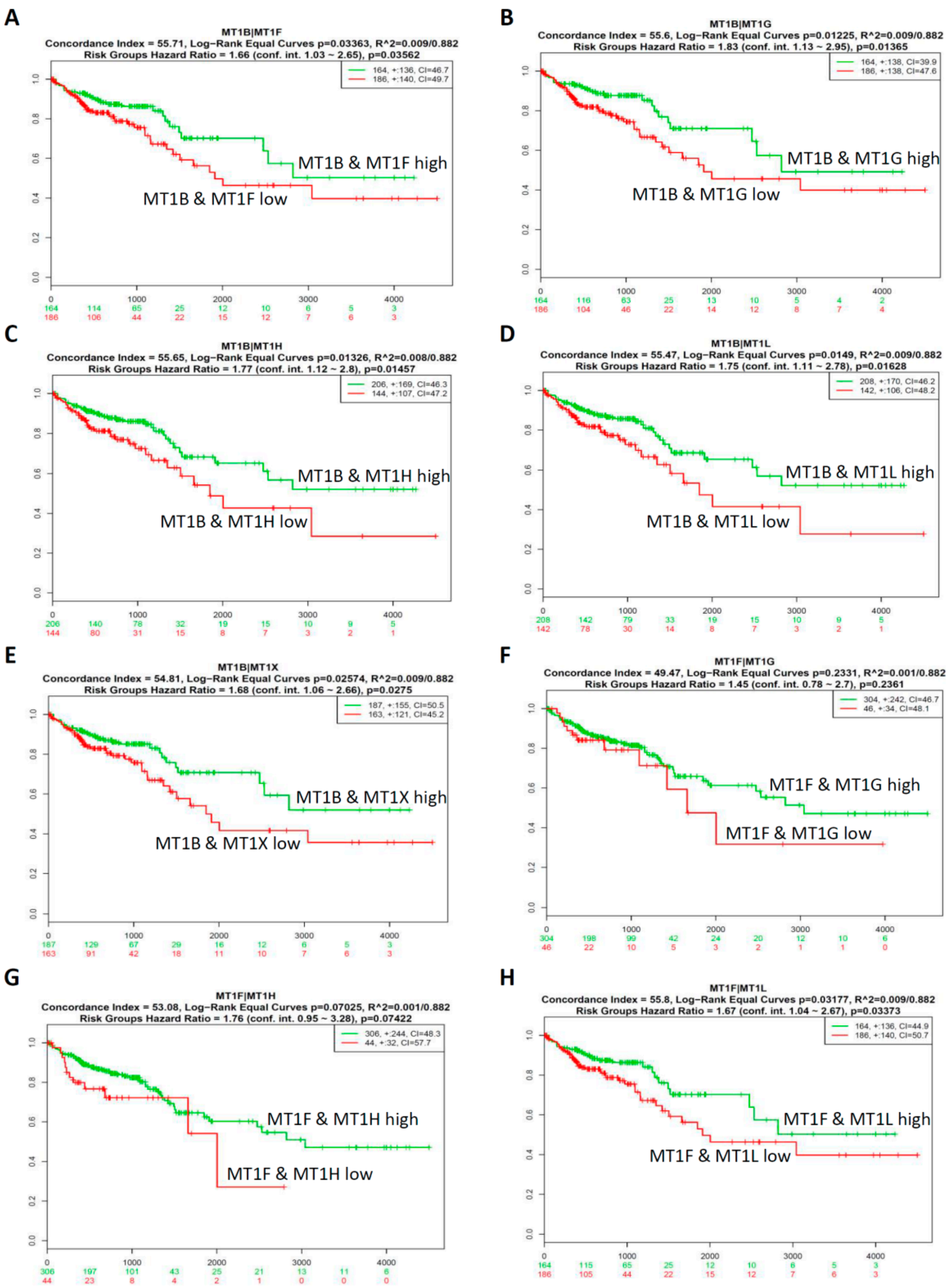

Supplementary Fig. 2

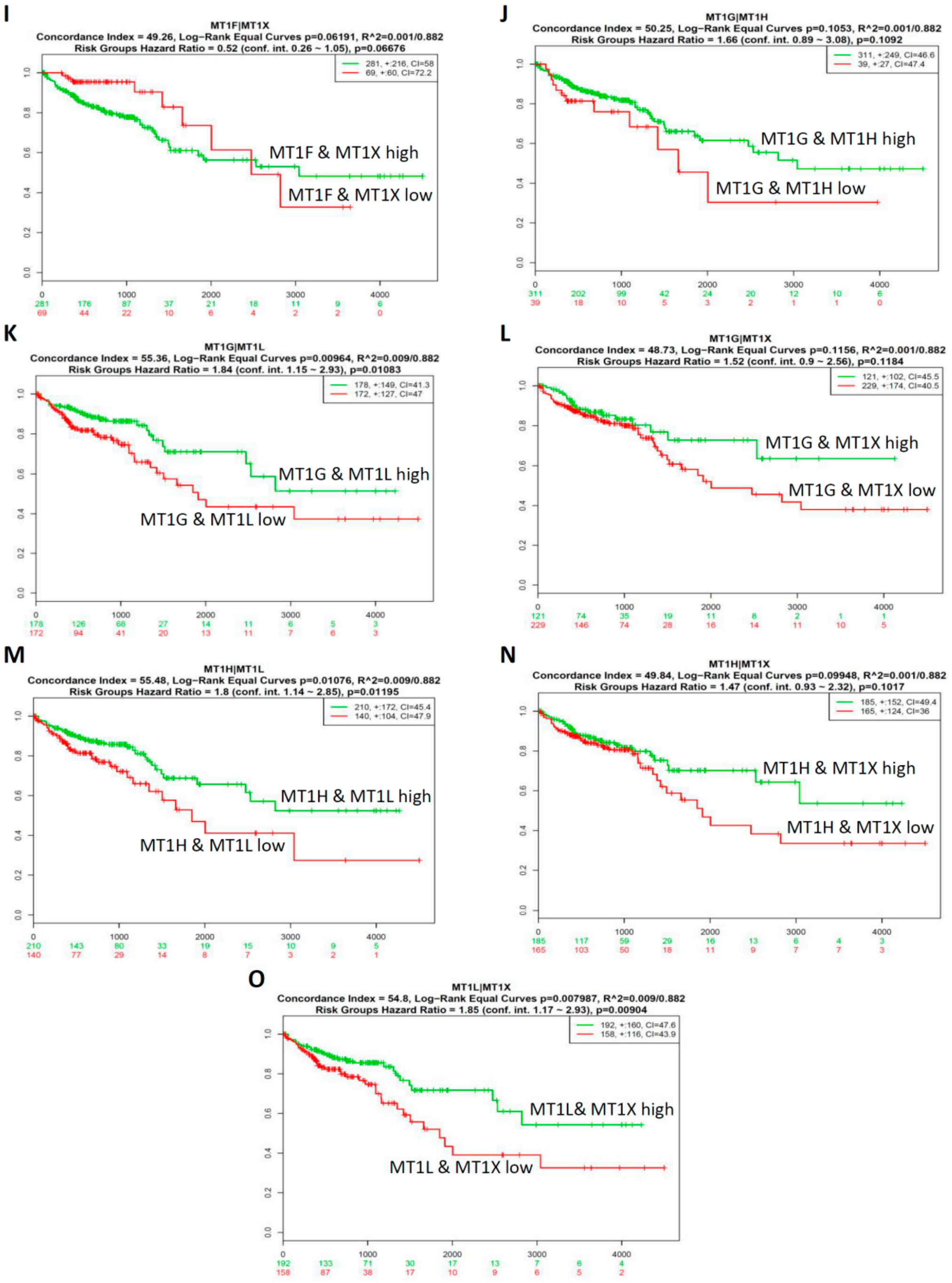

Supplementary Fig. 2 (Continue)

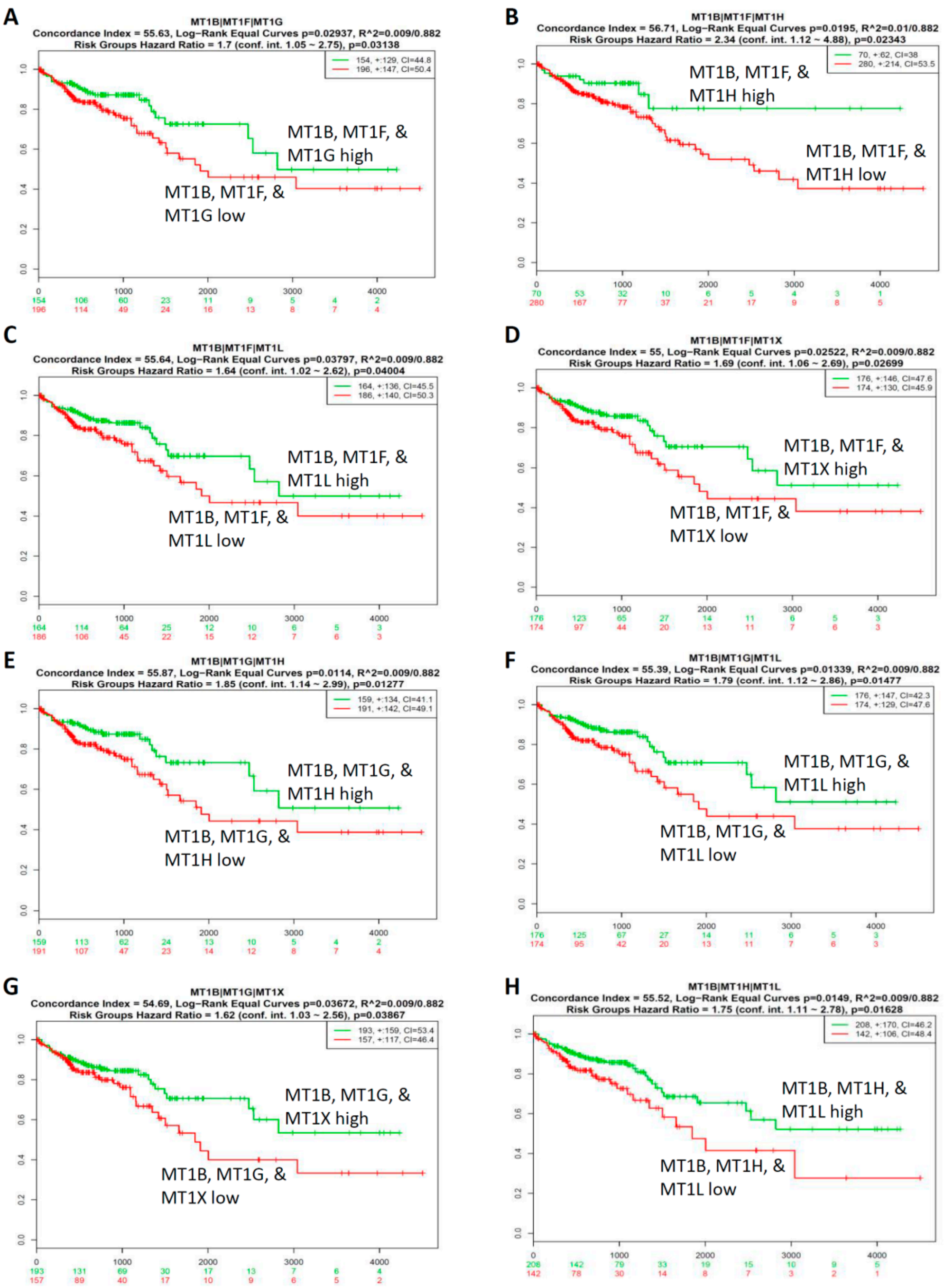

Supplementary Fig. 3

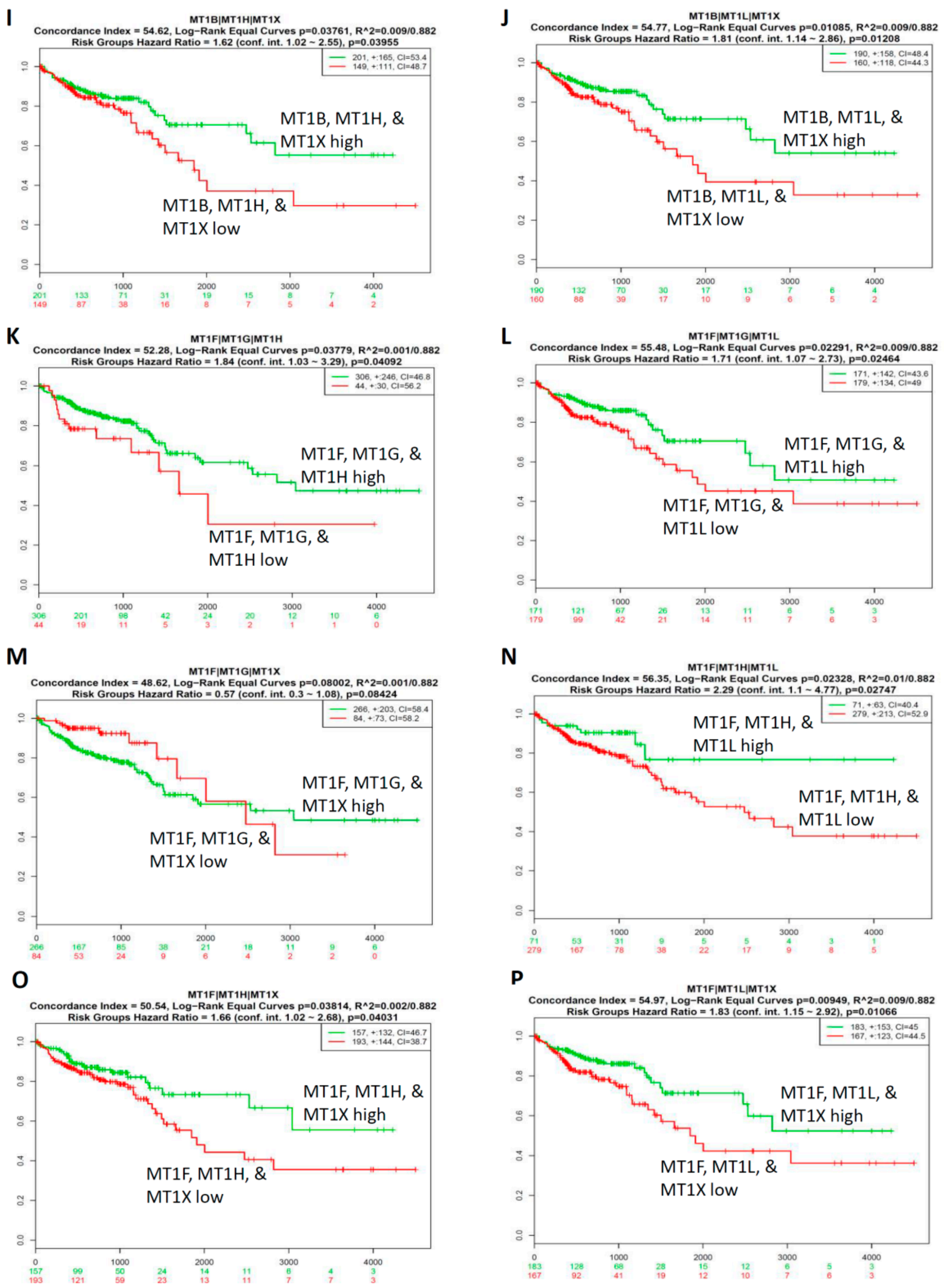

Supplementary Fig. 3 (Continue)

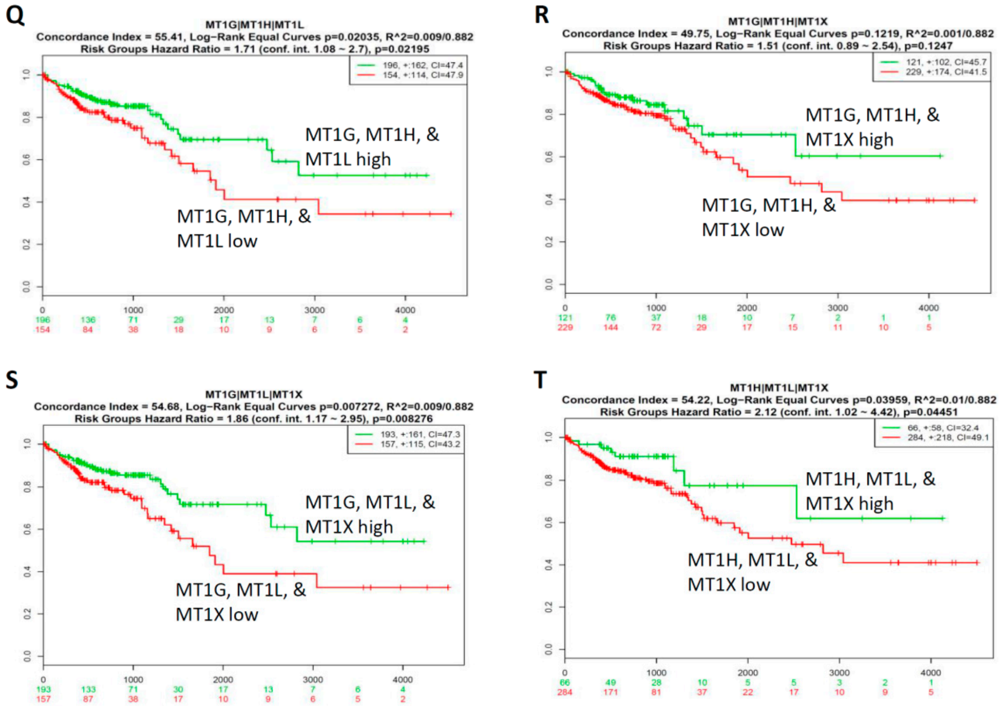

Supplementary Fig. 3 (Continue)

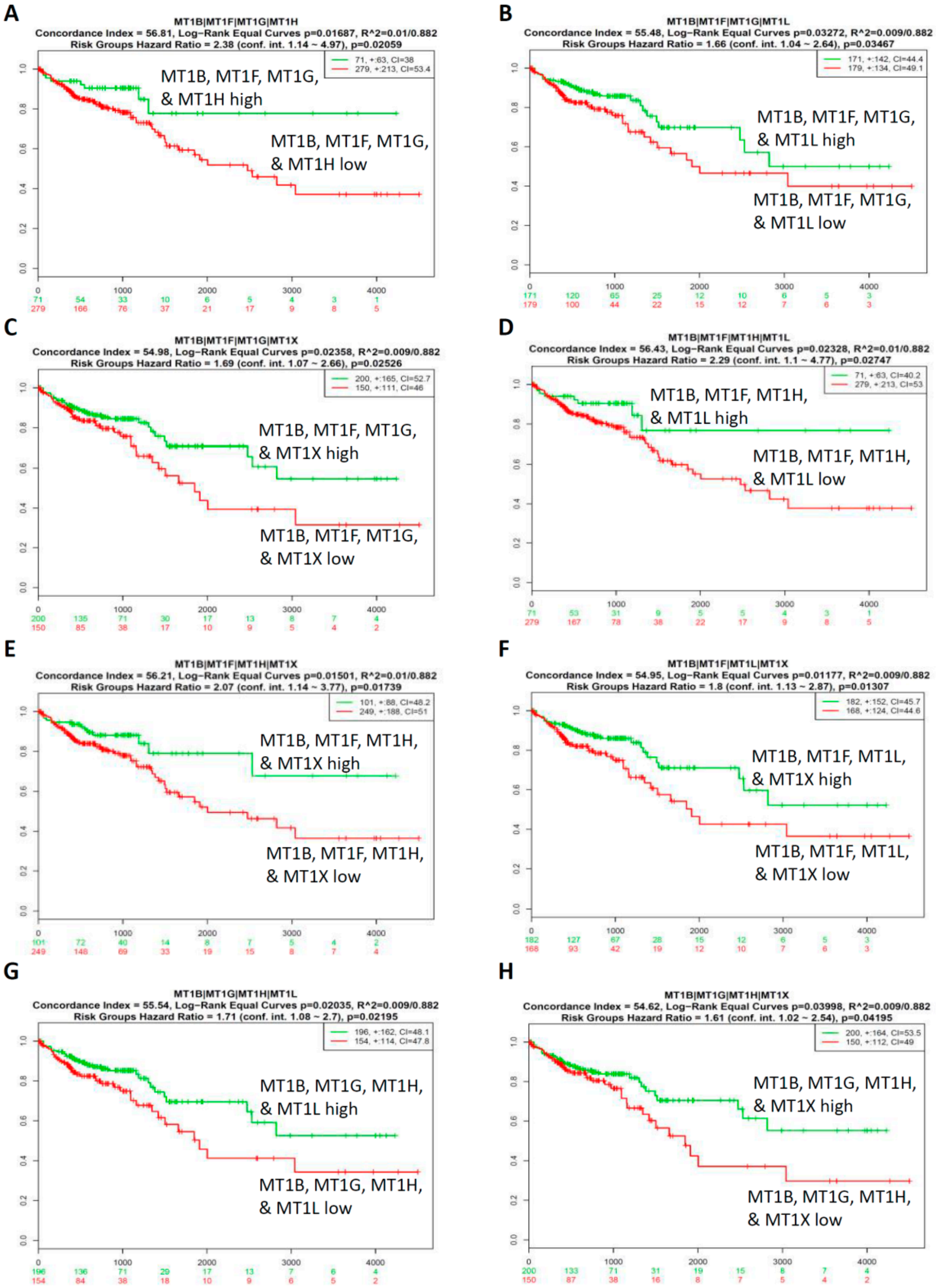

Supplementary Fig. 4

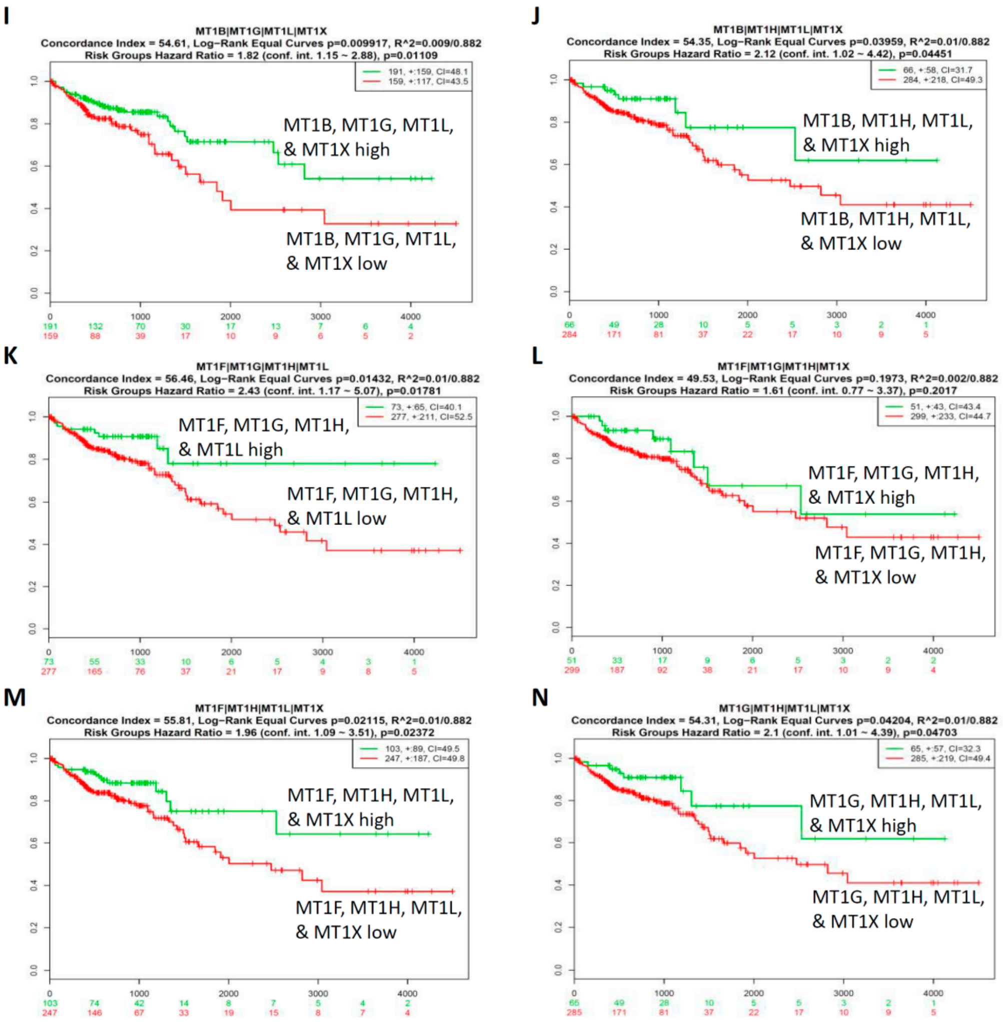

Supplementary Fig. 4 (Continue)

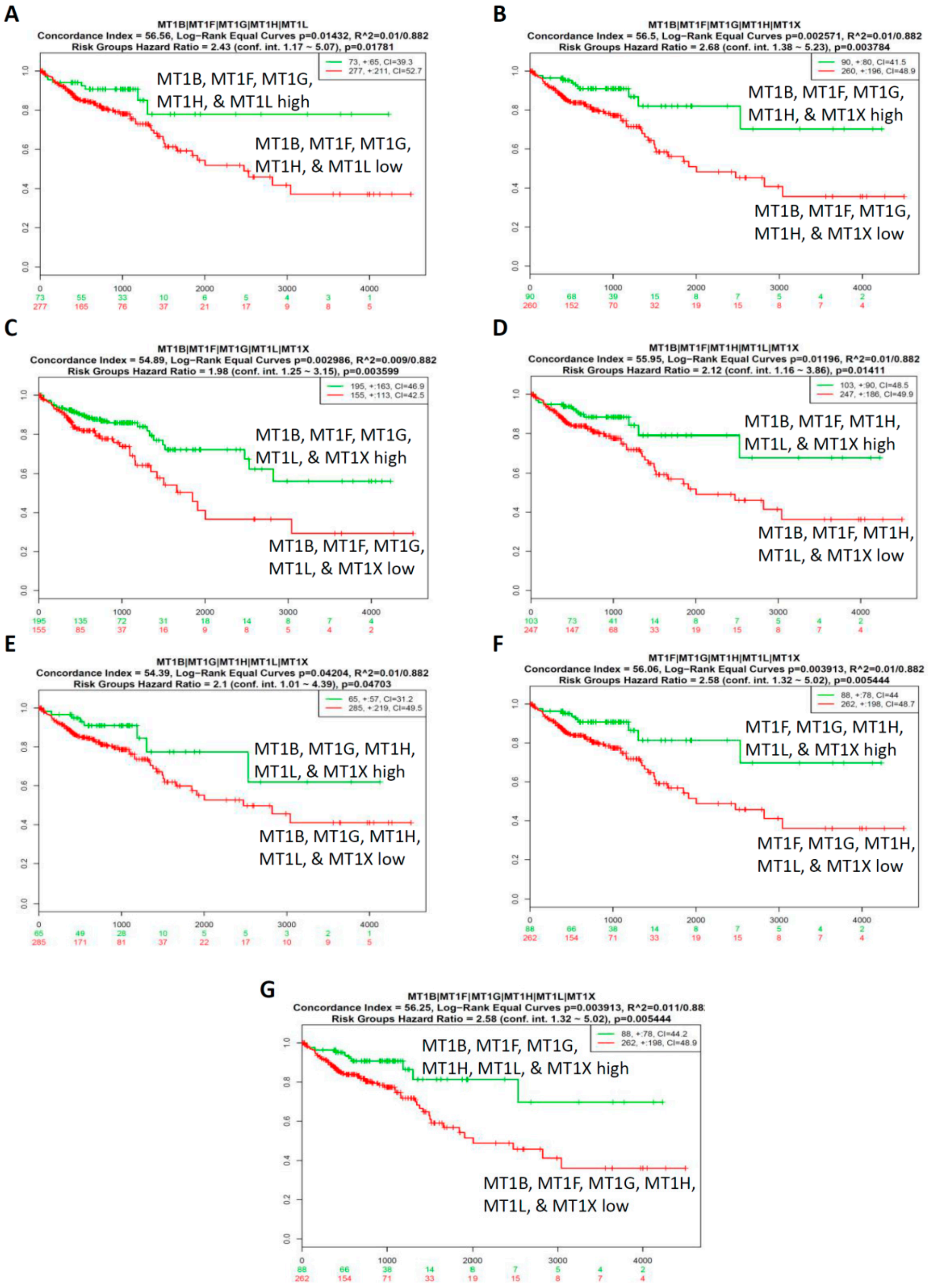

Supplementary Fig. 5
